# Accidental compression of the thoracic wall. Mechanical asphyxia rather than trauma is the main culprit

**DOI:** 10.1016/j.tcr.2024.101064

**Published:** 2024-06-06

**Authors:** Christos Voucharas, Angeliki Vouchara, Fani Tsolaki, Ioannis Tagarakis, Georgios Tagarakis

**Affiliations:** Medical School, Faculty of Health Sciences, Aristotle University of Thessaloniki, 54636, Greece

**Keywords:** Traumatic asphyxia, Thoracic compression, Thoracic trauma

## Abstract

We present three cases of traumatic asphyxia after thoracic compression. All victims were Caucasian males aged 22–50 years. One man was crushed by a truck trailer, another was crushed by an overturned vehicle, and the last was crushed by a large heavy stone slab. None of the patients survived the accident. There was no evidence of trauma or only minor trauma from the bones or vital organs of the thoracic cavity and abdomen.

## Highlights

• Casualty from traumatic asphyxia is an uncommon case of violent cessation of life.
• Contrary to the entire chest trauma, traumatic asphyxia has been rarely reported in literature.
• There is no evidence of trauma or there is only minor trauma.
• Traumatic asphyxia is usually suspected from the reported circumstances before an autopsy is performed.
• The impending death is dramatic and agonizing for the victim.

## Introduction

Blunt chest trauma is responsible for >15% of all trauma admissions to emergency departments worldwide and may result in injury to every thoracic structure [1].

Sustained external forces exerted on the chest or abdomen impede the performance of respiratory movements and obstruct the entry of air into the respiratory tracts, leading to mechanical asphyxia [2].

Asphyxia death resulting from compression of the chest wall may occur accidentally in cases of overcrowding, workplace accidents, traffic accidents, infants tightly wrapped in swaddling clothes, and even in a criminal act when the assailant sits on the victim's body [3].

Contrary to the entire chest trauma, traumatic asphyxia has been rarely reported in literature [4,5].

## Cases report

We present three cases of death due to traumatic asphyxia. They were Caucasian men aged 22, 40, and 50 years, who were crushed by a truck trailer, by an overturned vehicle, and a large heavy stone slab. None of the victims had a chance to be resuscitated at the scene of the accident.

The autopsy findings included edema, cyanosis, petechiae of the face, subconjunctival hemorrhages, ecchymosis, parchment-like impressions of the upper trunk, and subpleural petechiae.

The victim, who was crushed by the overturned vehicle, suffered a broken clavicle and ipsilateral ribs, as well as a hemothorax without pulmonary, myocardial, or abdominal organ contusion, and great vessel laceration.

## Discussion

The time required for the onset of traumatic asphyxia is typically 2–5 min after the chest compression [6]. Traumatic asphyxia is usually suspected from the reported circumstances before an autopsy is performed [7].

External examination and autopsy macroscopic findings are due to a huge increase in the central venous pressure and reversal of venous blood flow from the heart through the superior vena cava into the innominate and jugular veins of the head and neck, while arterial flow continues, resulting in capillary stasis and rupture [1]. Additionally, capillary damage from O2 deficiency, suction power in active breathing, and rupture of muscle fibers during the effort made to remove the pressing force and perform respiratory movements, all explain pinpoint hemorrhages from the viscera (thymus, pericardium, pleura, lungs) and thoracic muscles. These findings indicate a difficult and painful death [1].

If the victim survives compression of the chest, he/she may experience petechiae and cyanosis of the face, arms, and upper chest, facial swelling, conjunctival bleeding, confusional state, loss of consciousness, convulsions, visual disturbances, eye prolapse, loss of vision, ruptured viscera, fractured ribs, and renal failure approximately a week after the effect of the myoglobin of the compression-damaged muscles on the epithelium of the convoluted tubules [5].

The mortality rate of traumatic asphyxia is high; nevertheless, intense and immediate medical intervention - experienced or not - should be indicated because successful resuscitation can be expected even if ventilatory insufficiency, cardiac arrest, reversible severe chest trauma, and minor neurological trauma exist [8].

In survivors of a traumatic asphyxial episode, optimal management should focus on the early recognition of this entity based on classic physical signs and the history of injury. Supportive treatment should include oxygen uptake and stay in bed with raised bed head by 15–30°, cardiopulmonary resuscitation, assisted ventilation, closed thoracic drainage, blood infusion, fluid resuscitation with crystalloids to prevent renal complications of potential traumatic rhabdomyolysis, and care for brain edema with mannitol administration [9].

## Conclusion

Casualty from traumatic asphyxia is rare in cases of violent death. The impending death is difficult and agonizing for the victim. Autopsy findings are characteristic; however, the cause of death is essentially confirmed by the history of the injury, circumstances of the accident, and any existing witnesses. Demise results from functional airway obstruction rather than severe bone and visceral injuries, hemorrhage, or vital organ laceration.

## CRediT authorship contribution statement

**Christos Voucharas:** Writing – review & editing, Writing – original draft, Validation, Supervision, Methodology, Data curation, Conceptualization. **Angeliki Vouchara:** Writing – original draft, Validation, Software, Investigation, Formal analysis, Conceptualization. **Fani Tsolaki:** Writing – review & editing, Visualization, Validation, Data curation. **Ioannis Tagarakis:** Validation, Project administration, Methodology, Formal analysis, Conceptualization. **Georgios Tagarakis:** Writing – review & editing, Visualization, Methodology, Investigation, Conceptualization.

## Declaration of competing interest

No funding was received for the conception of this work or that could have influenced its outcome.

The authors report no conflict of interest. This research did not receive any specific grant from funding agencies in the public, commercial, or not-for-profit sectors.

No ethical approval is required/identifying information is not published.

No consent is required/identifying information is not published.
